# Comparison of two methods of visual magnification for removal of adhesive flash during bracket placement using two types of orthodontic bonding agents

**DOI:** 10.1590/2177-6709.21.6.043-050.oar

**Published:** 2016

**Authors:** Estefania Queiroga de Santana e Alencar, Maria de Lourdes Martins Nobrega, Fabio Roberto Dametto, Patrícia Bittencourt Dutra dos Santos, Fabio Henrique de Sá Leitão Pinheiro

**Affiliations:** 1 Student, Master Degree Program in Dentistry, Universidade Potiguar (Laureate International Universities), Natal, Rio Grande do Norte, Brazil; 2 Assistant professor, Universidade Potiguar, (Laureate International Universities), Master Degree Program in Dentistry and Faculty member of the Residence Program in Endodontics, Natal, Rio Grande do Norte, Brazil.; 3 Assistant professor, Department of Orthodontics, Universidade do Estado do Rio Grande do Norte (UERN), Caicó, Rio Grande do Norte, Brazil.; 4 Assistant professor, Department of Preventive Dental Science (Orthodontic Division), University of Manitoba, Winnipeg, Canada.

**Keywords:** Orthodontics, Composite resins, Lenses

## Abstract

**Objective:**

This study aimed to evaluate the effectiveness of two methods of visual magnification (operating microscope and light head magnifying glass) for removal of composite flash around orthodontic metal brackets.

**Material and Methods:**

Brackets were bonded in the center of the clinical crown of sixty well-preserved human premolars. Half of the sample was bonded with conventional Transbond XT (3M Unitek TM, USA), whereas the other half was bonded with Transbond TM Plus Color Change (3M Unitek TM, USA). For each type of composite, the choice of method to remove the flash was determined by randomly distributing the teeth into the following subgroups: A (removal by naked eye, n = 10), B (removal with the aid of light head magnifying glass, under 4x magnification, n = 10), and C (removal with the aid of an operating microscope, under 40x magnification, n = 10). Brackets were debonded and teeth taken to a scanning electron microscope (SS-x-550, Shimadzu, Japan) for visualization of their buccal surface. Quantification of composite flash was performed with Image Pro Plus software, and values were compared by Kruskal-Wallis test and Dunn’s post-hoc test at 5% significance level.

**Results:**

Removal of pigmented orthodontic adhesive with the aid of light head magnifying glass proved, in general, to be advantageous in comparison to all other methods.

**Conclusion:**

There was no advantage in using Transbond TM Plus Color Change alone. Further studies are necessary to draw a more definitive conclusion in regards to the benefits of using an operating microscope.

## RESUMO

**Objetivo:**

este estudo teve o objetivo de avaliar a eficácia de dois métodos de magnificação visual (microscópio cirúrgico e lupa de pala) para remoção da resina residual em torno de braquetes ortodônticos metálicos.

**Material e Métodos:**

os braquetes foram colados no centro da coroa clínica de 60 pré-molares humanos bem preservados. Metade da amostra foi colada com Transbond XT convencional (3M Unitek^TM^, EUA), enquanto a outra metade foi colada com Transbond^TM^ Plus Color Change (3M-Unitek^TM^, EUA). Para cada tipo de resina, a escolha do método para remover o resíduo foi determinada por meio da distribuição aleatória dos dentes nos seguintes subgrupos: A (remoção a olho nu, n = 10), B (remoção com a ajuda de lupa de pala, sob uma ampliação de 4x, n = 10) e C (remoção com auxílio de um microscópio cirúrgico, sob uma ampliação de 40x, n = 10). Os braquetes foram descolados e os dentes, levados a um microscópio eletrônico de varredura (SS-X-550, Shimadzu, Japão) para visualização de sua superfície vestibular. A quantificação da resina residual foi realizada por meio do *software* Image Pro Plus, onde os valores foram comparados utilizando-se o teste de Kruskal-Wallis e o teste *post-hoc* de Dunn, ao nível de significância de 5%.

**Resultados:**

a remoção da resina ortodôntica pigmentada com o auxílio da lente de aumento de pala provou, em geral, ser mais vantajosa, em comparação aos outros métodos testados.

**Conclusão:**

não houve vantagem em se usar a Transbond^TM^ Plus Color Change sozinha. Estudos adicionais são necessários para se chegar a uma conclusão definitiva sobre os benefícios da utilização de microscópio cirúrgico.

## INTRODUCTION

Although fixed appliances are effective in correcting malocclusions, plaque buildup (biofilm) tends to be an issue during prolonged orthodontic treatment. Some studies have demonstrated a clear association between biofilm and enamel demineralization.^1^ ^,^ ^2^ ^,^ ^3^ A linkage between biofilm and periodontal disease has also been established.^4^

Besides the regular use of oral hygiene aids by patients,^5^ ^,^ ^6^ ^,^ ^7^ most clinical procedures in Dentistry aim at avoiding or eliminating the creation of retentive areas or undercuts. Unfortunately, fixed appliances have many retentive sites which can be considered a risk factor for the development of caries and periodontal disease.

Similarly, composite flash around orthodontic brackets can also work as a type of retentive factor that goes often unnoticed. It has been demonstrated that unpolished and rough composite surfaces stimulate the accumulation of plaque and other debris.^8^ ^,^ ^9^ ^,^ ^10^

In addition, orthodontic bonding agents are known to be toxic to human gingival fibroblasts, thereby causing inflammation and gingival hyperplasia even in the presence of good oral hygiene.^11^ Therefore, careful removal of the bonding agent would reduce the risk of demineralization during orthodontic treatment.

To facilitate visualization of composite flash, the industry has developed a pigmented composite material of which pink color completely fades away during curing. The effectiveness of this type of material was assessed on typodonts in 2007, but the authors^12^ did not find it to be clinically advantageous. No method of visual magnification was used in this study while removing composite flash.

Another way to remove the flash consists in using visual resources, such as magnifying glasses or operating microscopes. The former was proposed in 2006 in a literature review^13^ on magnification devices that can be useful for Orthodontics. The operating microscope has been widely used in areas, such as Endodontics,^14^ Oral Surgery,^15^ and Periodontics.^16^ In Orthodontics; however, such methods have not been extensively put into practice. 

To date, studies in which clinical methods of magnification were used to help removing composite flash around orthodontic brackets are quite scarce. The following search strategy was run in PubMed database in August, 2012: (orthod* AND bracket AND |adhesive OR composite| AND |lens OR microscope OR magnification OR magnifying|). Only 75 articles were retrieved; out of these, only one^17^ studied the influence of magnification resources on the removal of orthodontic bonding agents, but its focus was on debonding. Alternative search strategies (“operating microscope AND orthodontic”; “loupe AND orthodontic”; “magnifying lens AND orthodontic”) also failed to retrieve relevant studies. 

The present study assessed the efficacy of two magnification resources (the light head magnifying glass and the operating microscope) in removing composite flash around orthodontic brackets. Both conventional and pigmented resins were also tested. The following null hypothesis (H0) was postulated: “In comparison to the naked eye, there is no statistically significant difference in the amount of composite flash when using either method of visual magnification, regardless of the type of bonding agent which was applied to the bracket mesh base (conventional or pigmented).”

## MATERIAL AND METHODS

### Sample selection

This study was reviewed and approved by the ethics committee (#008/2010) at Potiguar University (Laureate International Universities) in Natal, Brazil. Sixty-two human premolars that had been extracted for orthodontic or periodontal reasons were stored in a solution of 0.1% thymol at room temperature. These teeth were selected according to the following inclusion criteria: 1) no enamel defect, 2) no color alteration.

Sample size for each group was calculated based on a previous study,^12^ since it was the only report on this matter (n = 10).

### Sample preparation

Roots were removed cross-sectionally with a flexible diamond disk coupled to a low-speed hand piece. The cut was made at 3 mm from the cementoenamel junction. After sealing the root canals with composite (Flowable Restorative, 3M ESPE^TM^, USA), each selected tooth was positioned inside 20 x 10-mm PVC cylinders, maintaining the buccal surface centered and parallel to the base of the cylinder. Polyester resin was poured into the cylinders for partial inclusion of teeth, leaving the buccal surface exposed.

After 24 hours, the PVC cylinders were withdrawn and specimens stored in deionized water for 24 hours for rehydration. There was no intention to simulate the oral environment, as specimens were expected to be as clean as possible to allow accurate measurement of the composite-containing area. 

Slightly powdered vinyl gloves were used throughout the study. For complete removal of the powder, they were washed thoroughly with deionized water and later disinfected with 70% ethanol. 

### Teeth preparation

Dental prophylaxis with rubber cups and a mixture of deionized water and extra-thin pumice was performed for 10 seconds on the buccal surface of each tooth. The cups were replaced with new ones at every ten teeth. Specimens were washed for 30 seconds with an oil-free spray of deionized water and air. They were later dried out with oil-free air for 20 seconds.

The enamel was etched for 15 seconds with 37% phosphoric acid gel, then washed and dried, as described above. Etching was considered adequate based on the opaqueness of the surface. Shortly thereafter, the primer of Transbond XT (3M Unitek^TM^, USA) was applied with an extra fine brush. 

### Bracket bonding

Stainless steel 0.022 x 0.030-in Edgewise premolar brackets (Slim, Roth prescription, Morelli^TM^, Brazil) with a mesh base of 10.17 mm^2^ were used. The amount of resin to bond the brackets was standardized with a plunger-type dosimeter specially designed for this study.

Each bracket was centered on the buccal surface of each tooth using a self-locking bracket tweezer (Dentaurum, Germany). A pressure of 470 g was delivered against the bracket by a stainless steel needle adapted to the upper end of a mechanical press (Soft line, APEC, Brazil) in order to ensure maximum flash outflow. 

### Study groups

The combination of resin type (conventional or pigmented), method of visual magnification (light head magnifying glass or operating microscope) and gold standard control (naked eye) generated six experimental groups: Group A1 (conventional resin + naked eye), Group B1 (conventional resin + light head magnifying glass), Group C1 (conventional resin + operating microscope), Group A2 (pigmented resin + naked eye), Group B2 (pigmented resin + light head magnifying glass), and Group C2 (pigmented resin + operating microscope). To comprise each group, ten premolars (five maxillary and five mandibular) were randomly selected.

### Removal of composite flash

All brackets were held steady for 30 seconds by the opposite end of a bracket tweezer while a #5 dental explorer was used to scrape off the composite flash around and over the edges of the base.

During flash removal with the naked eye, a conventional dental reflector was used to illuminate specimens. The reflector position was previously standardized with regards to distance and angulation. The same source of light was used when bonding the teeth with the help of the light head magnifying glass (TK600, under 4x magnification, Lohcus - Comércio e Tecnologia em Saúde Ltda., Brazil). For the operating microscope group (DF Vasconcellos SA, Brazil, under 40x magnification), the built-in lamp served as reflector.

All bonding procedures were performed by two calibrated orthodontists with neither previous experience nor preference for a specific type of method, except for the gold standard (naked eye). Calibration sessions consisted in repeating each method until achieving consistency in terms of composite flash removal within the time span of 30 seconds. To avoid performance bias, the order with which each method was carried out was determined at random.

Following removal of the composite flash, samples were light-cured for 40 seconds (20 seconds mesial and 20 seconds distal) by a LED device (Radii Plus SDI, Brazil). The light unit tip was angulated 45 degrees and held as close as possible to the tooth surface. Debonding pliers were carefully used to successfully remove all the brackets in a way that the underlying composite was left intact, taking the shape (imprint) of the bracket mesh base. This helped the authors to develop a method to measure the composite flash area as it is explained below. 

### SEM preparation

Specimens were taken to an incubator at 80^o^C for 20 minutes with the purpose of melting down the polyester resin, thereby facilitating its removal. Subsequently, teeth were coated with a layer of gold (approximately 150 Angstroms) with the aid of Shimadzu IC-50 equipment. The coating procedure lasted 3 minutes at a current of 6 mA. The next step consisted of analyzing the images on a scanning electron microscope (SSX-550, Shimadzu, Japan) of which settings were adjusted for back scattering function with acceleration voltage of 20 kv. This function enables compositional contrast between the surface of the tooth (hydroxy apatite) and the resin (polymer).

**Figure 1:**
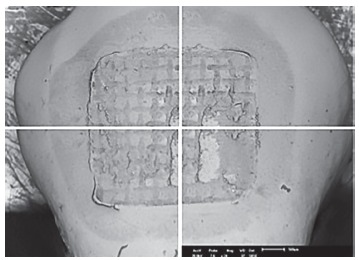
Image registration by quadrant (20x magnification each).

Magnification at 32x, although used in a previous study,^12^ did not allow full visualization of bracket surroundings. In order to enable analysis of the entire buccal surface on a single photograph, images of the four quadrants were individually captured and later assembled (Fig 1).

### Quantification of composite flash

To serve as control and help distinguishing the difference between composite and technical artifacts, the buccal surface of two teeth was demarcated and the following treatment modalities applied: upper left quadrant = etching; upper right quadrant = etching + bonding agent; lower left quadrant = etching + bonding agent + conventional resin; and lower right quadrant = etching + bonding agent + pigment resin (Fig 3).

**Figure 2:**
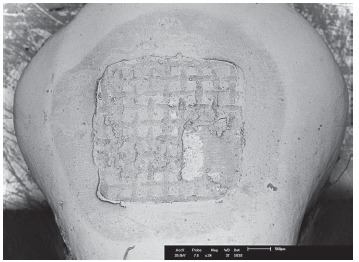
The entire image assembled by the superimposition of the four quadrants.

**Figure 3:**
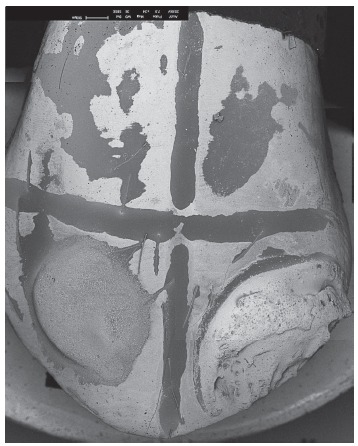
Control sampl: In upper left quadrant, etching; in upper right quadrant, etching + bonding agent; in lower left quadrant, etching + bonding agent + conventional resin; in lower right quadrant, etching + bonding agent + pigment resin.

**Figure 4:**
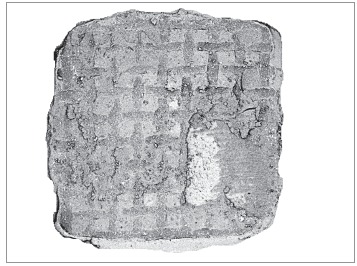
After removal of tooth structure.

**Figure 5:**
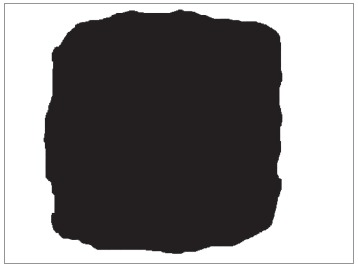
After increasing the contrast with the Image Pro Plus software.

To isolate the area where only composite was present, any image suggestive of tooth structure (brighter areas) was removed (Fig 4). Contrast was adjusted by Image Pro Plus software (Fig 5) of which calibration was made possible by means of the scale available in each picture. The entire remaining dark area was measured in mm^2^. In order to quantify only the area of ​​interest (resin around the bracket), it was necessary to mathematically subtract the value corresponding to the underlying resin (bracket mesh base = 10.17 mm^2^).The operator in charge of carrying out these measurements was not aware of the hypotheses being tested. 

### Statistical analysis

The values ​​of the areas in each group were stored in a datasheet of SPSS (Statistical Package for the Social Sciences, SPSS Inc, Chicago, IL, version 9.0 for Microsoft Windows) for statistical analysis. Shapiro-Wilk test was used to analyze sample distribution. Given the absence of normal distribution in two groups (B2 and C2), intergroup comparison was performed with Kruskal-Wallis test followed by Dunn’s post-hoc test for multiple comparisons. In all analyses, significance level was set at 5%.

## RESULTS

Descriptive statistics containing sample size, median, minimum and maximum values​​, 25^th^- 75^th^ percentiles, and the interquartile range is available in Table 1. Kruskal-Wallis test at 5% with a KW statistic of 32.604 (corrected for ties) and 5 degrees of freedom detected statistically significant difference between medians (*p*< 0.0001). Dunn’s test for multiple comparisons identified five pairs of groups with statistically significant difference (*p* ≤ 0.05) (Table 2).

**Table 1:** Descriptive statistics

Groups	n	Median	Min-Max	25th. - 75th.	Interquartile range
		(mm^2^)	(mm^2^)	percentiles	
A1 Conv. + eye	10	4.76	1.40 - 8.68	3.62 - 6.23	2.62
B1 Conv. + Light head	10	6.94	3.53 - 14.50	5.38 - 8.82	3.44
C1 Conv. + OM	10	5.95	0.72 - 7.73	2.05 - 7.56	5.51
A2 Pigm. + eye	10	2.93	0.00 - 4.16	1.59 - 3.75	2.15
B2 Pigm. + Light head	10	0.43	0.00 - 2.30	0.11 - 1.26	1.15
C2 Pigm. + OM	10	1.11	0.00 - 6.98	0.57 - 2.74	2.17

Conv = Conventional orthodontic adhesive; Pigm = Pigmented orthodontic adhesive; OM = Operating microscope.

**Table 2:** Intergroup comparison.

Intergroup Comparison	Median Difference	Flash area	Mean Rank Difference	P value
	(1st. - 2nd.)			
A1 (Conv. + eye) vs A2 (Pigm. + eye)	1.83	A1 > A2	13.867	ns
A1 (Conv. + eye) vs B1 (Conv. + Light head)	-2.18	B1 > A1	-9.633	ns
A1 (Conv. + eye) vs B2 (Pigm. + Light head)	4.33	A1 > B2	27.567	**
A1 (Conv. + eye) vs C1 (Conv. + OM)	-1.19	C1 > A1	0.3667	ns
A1 (Conv. + eye) vs C2 (Pigm. + OM)	3.65	A1 > C2	18.967	ns
A2 (Pigm. + eye) vs B1 (Conv. + Light head)	-4.01	B1 > A2	-23.500	*
A2 (Pigm. + eye) vs B2 (Pigm. + Light head)	2.5	A2 > B2	13.700	ns
A2 (Pigm. + eye) vs C1 (Conv. + OM)	-3.02	C1 > A2	-13.500	ns
A2 (Pigm. + eye) vs C2 (Pigm. + OM)	1.82	A2 > C2	5.100	ns
B1 (Conv. + Light head) vs B2 (Pigm. + Light head)	6.51	B1 > B2	37.200	***
B1 (Conv. + Light head) vs C1 (Conv. + OM)	0.99	B1 > C1	10.000	ns
B1 (Conv. + Light head) vs C2 (Pigm. + OM)	5.83	B1 > C2	28.600	**
B2 (Pigm. + Light head) vs C1 (Conv. + OM)	-5.52	C1 > B2	-27.200	**
B2 (Pigm. + Light head) vs C2 (Pigm. + OM)	-0.68	C2 > B2	-8.600	ns
C1 (Conv. + microsc.) vs C2 (Pigm. + microsc.)	4.84	C1 > C2	18.600	ns

Conv. = Conventional orthodontic adhesive; Pigm = Pigmented orthodontic adhesive; OM = Operating microscope; ns = non-significant (*p* > 0.05).

*Statistically significant when *p* ≤ 0.05; **Statistically significant when *p* ≤ 0.01; *** Statistically significant when *P* ≤ 0.001.

According to data presented in Table 2, only the combination of pigmented composite and light head magnifying glass (B2) yielded a result that was superior to the combination of conventional composite and naked eye (A1). The combination of pigmented composite and naked eye (A2) was more beneficial than the combination of conventional composite and light head magnifying glass (B1). In comparison to the combination of conventional composite and light head magnifying glass (B1), both the operating microscope (C2) and the light head magnifying glass(B2) performed better when associated with the pigmented composite. 

## DISCUSSION

The pigmented composite Transbond TM Plus Color Change (3M Unitek^TM^, USA) is a good example of a material developed to facilitate visualization of composite flash during bracket placement. The first study^2^ to test this material was published in June 2004. By the time this manuscript was written, only two more similar articles^3^ ^,^ ^18^ had been published, but none of them specifically addressed the advantages of pigmented orthodontic adhesives to facilitate the removal of flash around orthodontic brackets. 

Considering that the amount of composite left around orthodontic brackets tends to be quite significant, it is surprising that orthodontists are not so much concerned about it. In the present study, an area of up to 6.94 mm^2^ of remaining composite was observed. A large amount of composite flash was also described elsewhere.^12^

Busy clinical schedules and increased effort in obtaining the most ideal bracket positioning may be two main reasons to explain why most orthodontists tend to overlook composite flash. Also, the color of conventional orthodontic adhesives matches quite well the color of enamel. This can certainly produce the false impression that flash was completely removed.

It was once thought that it could be advantageous to leave a certain amount of resin around brackets to seal any gaps between them and the enamel.^19^ ^,^ ^20^ ^,^ ^21^ However, the study by Farrow et al^21^ could not confirm this hypothesis either when using fluid resins or composites reinforced with inorganic particles.

The present study aimed to investigate the most effective method to remove composite adjacent to orthodontic brackets. Besides evaluating the influence of the incorporation of pigments, this study also evaluated the advantages of two different methods of visual magnification: the operating microscope and the light head magnifying glass.

The method to measure the area of composite flash was based on the study by Armstrong et al.^12^ Unlike magnification used in their study, which was 32x, the present authors preferred to work with a magnification of 20x per quadrant, and then assemble the complete picture of the tooth by overlapping the four quadrants. This change proved necessary because the magnification of 32x did not allow for full visualization of the buccal surface containing the bracket.

Time spent on removal of flash composite was stringently controlled. In addition to avoiding performance bias, this was also helpful in assessing the clinical viability of each method. For orthodontists, turning a simple bracket placement procedure into a complex and time-consuming operation would not be economically feasible. In the present study, as far as flash removal with the naked eye is concerned, there was no advantage in using pigmented composite. This finding is consistent with a previous report.^12^

However, removal of pigmented resin with the aid of the light head magnifying glass appeared to be the most advantageous method. Besides being more effective than removing conventional composite with or without magnification, such low-cost and user-friendly method yielded a result very similar to removing pigmented composite with the aid of the operating microscope (Tables 1 and 2). In the era of excellence in Orthodontics, these data suggest that the combination of pigmented resin with light head magnifying glass should be encouraged when orthodontic assistants are removing composite flash. Orthodontists could then check for bracket placement immediately thereafter with no need for visual aids. 

In fact, a much better outcome was expected from the operating microscope. Both types of composites (conventional and pigmented), when removed with the aid of the operating microscope, performed very similarly to the removal of conventional composite with the naked eye (Table 2). However, it may be inaccurate to state that the operating microscope does not add precision to composite flash removal. In order to investigate this, methods without any time restriction could be of great value, as this might have contributed to the poor performance of the operating microscope. No matter how calibrated the operator may be, a longer bonding time will usually be necessary whenever using an operating microscope. This happens because the movements of the dental explorer require frequent focal adjustments. In addition, considering that bracket positioning is quite an art which requires full visualization of the tooth, and that patients may move during the procedure, it is unlikely that the operating microscope will gain much popularity in the orthodontic community.

The identification of a simple and low-cost method, such as the combination of pigmented composite and light head magnifying glass, is in itself something that deserves consideration. Assessing the influence of the time spent on adjusting the operator’s position to the indirect vision transmitted by the operating microscope can finally decide whether it is advantageous to use this type of technology in Orthodontics. 

## CONCLUSIONS

1) The removal of a pigmented orthodontic adhesive with the aid of the light head magnifying glass proved, in general, to be advantageous compared to all other methods tested.

2) It was not possible to accurately assess the benefits from the combination of a pigmented composite and the operating microscope, thus eliciting the importance of further studies designed to adapt its technical requirements to the orthodontic clinical setting.
